# Prevalence of sickle cell anemia in Africa: A protocol for a meta-analysis of existing studies

**DOI:** 10.1371/journal.pone.0321535

**Published:** 2025-04-21

**Authors:** Bwambale Jonani, Emmanuel Charles Kasule, Herman Roman Bwire, Otema Ricky, Joel Fredrick Arturo, Matsiko Livingstone, Nassinde Esther, Namirembe Esther, Namale Joanitah, Lillian Nabbanja Naava, Ssebulime Stephen, John Bosco Mundaka

**Affiliations:** 1 Laboratory department, Sebbi Hospital, Wakiso district, Uganda; 2 National STI Control Unit Mulago Hospital, STI/Skin Clinic - Old Mulago Hospital, Kampala, Uganda; 3 Department of Nursing, Sebbi Hospital, Wakiso district, Uganda; 4 Insurance and Client care department, Sebbi Hospital, Wakiso district, Uganda; 5 Obstetrics and Gynecology department, Sebbi Hospital, Wakiso district, Uganda; University of Illinois at Chicago, UNITED STATES OF AMERICA

## Abstract

**Background:**

Sickle cell anemia (SCA) rank 11th among all causes of mortality in Sub-Saharan Africa, with the region accounting for 75% of global cases. Inconsistent diagnostic methods and country-specific data gaps hinder current prevalence estimates. This systematic review and meta-analysis aim to provide pooled prevalence estimates and examine geographic and temporal trends in SCA.

**Objectives:**

This systematic review and meta-analysis aim to determine the pooled prevalence of SCA across Africa and analyze its geographic and temporal distribution patterns by synthesizing data from existing studies.

**Methods:**

We will search four major databases: PubMed, Google Scholar, BASE, and Scopus, for studies on SCA prevalence in Africa published between 1994 and 2024. We will use Zotero to remove duplicates and screen titles and abstracts. We will assess the methodological quality with the JBI critical appraisal checklist for studies reporting prevalence data and extract data using a tested MS Excel form from studies with low to moderate bias. We will use random-effects meta-analysis to calculate pooled prevalence estimates and conduct subgroup analyses for heterogeneity. We will evaluate publication bias with funnel plots and Egger’s test, using trim-and-fill analysis if asymmetry is detected. The strength of evidence will be assessed using the AMSTAR (A Measurement Tool to Assess systematic Reviews).

**Expected Results:**

We expect to find significant variations in SCA prevalence across different regions and age groups, reflecting underlying environmental, diagnostic and methodological factors. We aim to identify any shifts in SCA prevalence and distribution patterns, which could inform future public health strategies and interventions

**Discussion:**

Our analysis will reveal the pooled prevalence of SCA in Africa, influencing diagnostic, environmental, and methodological factors, guiding targeted public health interventions.

**Registration:**

Our meta-analysis protocol was registered with the Open Science Framework (OSF) on 8^th^ January, 2025. https://doi.org/10.17605/OSF.IO/M8JXV

## Introduction

Sickle Cell Anemia (SCA) arises from a point mutation in the HBB (hemoglobin subunit beta) gene, which encodes the β-globin subunit of hemoglobin [1]. Inheriting one sickle hemoglobin (HbS) mutation results in sickle cell trait (SCT), while inheriting two mutations, including at least one HbS, leads to Sickle Cell Anemia. While SCT, an evolutionary adaptation against severe malaria [2,3] is largely asymptomatic, SCA manifests as a severe, lifelong condition that significantly affects quality of life and reduces lifespan.

SCA is characterized by the abnormal sickling of red blood cells (RBCs), leading to microvascular occlusion and impaired oxygen delivery [4]. This triggers acute and chronic complications, including pain crises, stroke, renal dysfunction, and acute chest syndrome (ACS) [5,6]. Patients also face heightened infection risks, complications during pregnancy, and elevated maternal mortality [7,8]. These complications impose substantial medical, social, and economic burdens, particularly in low-resource settings.

The global distribution of SCA correlates with malaria-endemic regions, with Africa accounting for approximately 75% of global cases [9,10]. As shown by the World Health Organization (WHO), in sub-Saharan Africa (SSA), SCA is ranked 11^th^ among all-cause mortality, with the prevalence of the sickle-cell gene varying from 10% to 40% [11]. Between 2000 and 2021, newly diagnosed SCA cases increased worldwide at a rate of 382 per 100,000 live births, from 453,000–515,000, with the highest rate increase in SSA[11]. This high prevalence highlights the continent’s significant challenges in addressing SCA due to limited healthcare resources, inadequate diagnostic facilities, and inconsistent access to preventive care, particularly in rural and underserved areas [12].

Despite numerous studies on SCA prevalence, existing estimates vary widely due to differences in methodologies, and geographic focus. Many studies rely solely on clinical diagnosis or use inconsistent laboratory methods, introducing variability in case identification. Additionally, most studies are localized, limiting generalizability and leaving significant geographic gaps. Small sample sizes and selection biases further hinder statistical power and precision. Temporal trends in SCA prevalence are poorly understood, as factors like urbanization, improved medical care, and shifting population demographics complicate the interpretation of data.

A meta-analytic approach is essential to address these limitations. By synthesizing data across studies, accounting for methodological heterogeneity, and applying robust statistical techniques, this review aims to provide comprehensive prevalence estimates of SCA in Africa.

## Materials and methods

### The aim, design and setting of the study

This meta-analysis aims to answer two primary research questions:

1. What is the estimated prevalence of Sickle Cell Anemia across Africa?
2. What are the geographic and temporal trends in SCA prevalence across the African continent?

We have designed this study following the Preferred Reporting Items for Systematic Review and Meta-Analysis Protocols (PRISMA-P) 2015 guidelines (S1 File). The review will synthesize data from studies conducted in both urban and rural settings across Africa, incorporating various diagnostic approaches and population groups.

### Eligibility criteria

#### Study characteristics.

We will include studies published in any language between 1994 and 2024 that report primary data on SCA prevalence in African populations. Given the immense linguistic diversity of Africa, where many countries primarily use languages other than English, we will include studies in all languages to ensure comprehensive data collection. For studies published in languages other than English, we will utilize translation services to ensure accurate data extraction and analysis. This approach will mitigate the risk of bias and ensure that the data reflects the true prevalence of SCA in various linguistic and cultural contexts. The 30-year timeframe allows for comprehensive temporal trend analysis while maintaining contemporary relevance. We will consider all study designs that report prevalence data

#### Population.

The review will encompass studies focusing on all age groups, including newborns, children, and adults, from all African countries. We will include both community-based and hospital-based studies.

#### Diagnostic methods.

We will include studies that specified the prevalence of hemoglobin genotype as HBSS. Studies must have utilized one or more of the following diagnostic methods: solubility tests, rapid sickling tests (POCT), hemoglobin electrophoresis, high-performance liquid chromatography (HPLC), isoelectric focusing, or DNA testing. Studies relying solely on clinical diagnosis or microscopy without further laboratory confirmation will be excluded to ensure diagnostic reliability.

### Information sources and search strategy

We will search four major electronic databases: PubMed, Google Scholar, Bielefeld Academic Search Engine (BASE), and Scopus. We developed search strings using medical subject headings (MeSH) from the Yale MeSH Analyzer tool. The PMIDs 26833239, 31451441, and 22958698 fed into the MeSH Analyzer generated terms; “sickle cell,” “prevalence,” “epidemiology,” “burden,” and “Africa.” We varied and combined these terms to create the initial search string in PubMed Central, which was reviewed and refined by Bwambale Jonani (BJ) and Herman Roman Bwire (HRB), then validated for ability to retrieve eligible studies. We converted the string for use in Google Scholar, BASE, and Scopus using the Systematic Review Accelerator – Polyglot Search Translator [13]. The closest match for Google Scholar was the Web of Science string. Peer reviewers, including Emmanuel Charles Kasule (ECK), Namale Joanitah (NJ), Namirembe Esther (NE), Nassinde Esther (NE), Otema Ricky (OR), Joel Fredrick Arturo (JFA), and John Bosco Mundaka (JBM), proofread the strategy. We accessed Scopus and Google Scholar via Harzing’s Publish or Perish software [14]. BJ and HRB independently conducted the search. Search strings and tables for each database are in Supplementary Information, S2 File. We will examine reference lists of included studies and relevant reviews for literature saturation

### Study selection and data management

Before screening, all reviewers will pilot screen a small sample of records to ensure consistency in applying eligibility criteria. We will manage study selection using Zotero citation management software (version 7.0.11, 64-bit), following a systematic approach to record screening and selection. Three reviewers (BJ, ECK, and HRB) will independently screen titles and abstracts against predefined eligibility criteria. Inter-reviewer agreement will be assessed using Cohen’s kappa, and group discussions will address any inconsistencies and clarify screening criteria (S3 File**).** Studies meeting initial criteria will proceed to full-text review by six reviewers (BJ, ECK, NJ, NE, JFA, and HRB) for final inclusion. Consensus discussions will resolve disagreements at the full-text review stage, with JBM and Ssebulime Stephen (SS) serving as arbitrators if needed. Bibliographic data of screened, excluded, and included records will be exported and shared as RIS files. The final dataset summarizing screening decisions will be shared in an open repository on the Open Science Framework and Figshare as an MS Excel File (S4 File)

### Data extraction and quality assessment

All six reviewers (BJ, ECK, NJ, NE, JFA, and HRB) will undergo training on the data extraction form (S5 File**).** They will jointly extract data from each included study, while another pair of reviewers (OR and NE) will validate a random 10% sample to ensure accuracy. The extraction form will capture:

1. Study characteristics (authors, publication year, country, study design)
2. Population demographics (age distribution, gender ratio, setting)
3. Methodological features (sampling strategy, diagnostic methods)
4. Outcome measures (prevalence data, case numbers, denominators)
5. Quality indicators (response rates, missing data handling)

We will assess the methodological quality of included studies using the Joanna Briggs Institute (JBI) critical appraisal checklist for prevalence studies. This tool evaluates key domains such as sampling methodology, case definition, measurement reliability, and statistical analysis. Only studies with low and moderate risk of bias will be included in the meta-analysis. Extracted data will be organized and shared as an MS Excel file on both Figshare and the Open Science Framework.

### Data synthesis and statistical analysis

#### Planned data transformations.

To ensure consistency across studies, we will standardize raw prevalence data by converting all values to proportions. When subgroup-specific prevalence is reported, we will adjust to recalculate the overall population prevalence where possible. Countries will be categorized into standardized African regions: North, West, Central, East, and Southern Africa, to facilitate regional subgroup analyses. Diagnostic methods used in the studies will be grouped into broader categories for comparison: Biochemical Methods (Solubility Tests, Rapid Sickling Tests (POCT)), Electrophoretic Techniques (Hemoglobin Electrophoresis, Isoelectric Focusing), Chromatographic Methods (High-Performance Liquid Chromatography (HPLC)), Microscopic Analysis (Morphological Analysis (Microscopy)), and Molecular Methods (DNA Tests). Age will be reclassified into standardized groups: <1 year, 1–4 years, 5–14 years, 15–49 years, ≥50 years) to enable subgroup analysis. Additionally, each study will be grouped according to its publication date (1994–1999, 2000–2004, 2005–2009, 2010–2014, 2015–2019, 2020–2024). For longitudinal studies, the midpoint of the data collection period will be used for classification

#### Synthesis plan.

If the statistical heterogeneity of all included studies I² ≤ 50%, and the Cochran’s Q test is non-significant, we will proceed to meta-analysis. High heterogeneity (I² > 50%) will prompt subgroup analyses to explore variability based on factors such as study design, population type, diagnostic method, and healthcare setting. Groups of studies with fewer than five studies per subgroup will undergo narrative synthesis rather than meta-analysis. The pooled prevalence of SCA will be calculated using a random effects model, accounting for variability across studies. Heterogeneity will be assessed using I² statistics and Cochran’s Q test, and forest plots will be generated to visually display individual study estimates and pooled effects. Publication bias will be assessed using funnel diagrams and Egger’s test, with trim-and-fill methods used to estimate the impact of missing studies due to publication bias and adjust the pooled prevalence estimates accordingly. Subgroup analyses will be conducted by geographic region, age group, study period, to assess the geographical, age group variations and temporal trends in the prevalence of SCA over the last three decades.

#### Sensitivity testing.

Sensitivity analyses will be conducted to assess the robustness of the synthesis results. Each study will be systematically excluded one at a time to observe the impact on the pooled prevalence estimate. Studies that significantly alter the pooled estimates upon exclusion will be documented to evaluate their influence. To address the studies significantly influencing the pooled prevalence estimates, the characteristics of these studies will first be investigated, focusing on aspects such as study design and sample size, to understand why they have a disproportionate impact. Following this, the pooled prevalence will be recalculated after excluding the influential studies, and the adjusted estimates will be compared to the original results. We will compare the confidence intervals (CIs) of the original pooled prevalence estimate and the recalculated estimate. If the CIs overlap substantially, it will indicate that the exclusion of the studies had a minimal impact on the findings. If the CIs are non-overlapping, it will suggest a significant difference.

### Quality of evidence assessment

We will assess the overall quality of evidence using AMSTAR (A Measurement Tool to Assess systematic Reviews). This tool evaluates the methodological quality of systematic reviews based on the comprehensiveness of the literature search, the appropriateness of the study selection criteria, the transparency of the data extraction process, quality of the included studies and the synthesis of the findings

### Ethical considerations

As this is a meta-analysis of existing studies, it does not involve direct human subjects and therefore does not require ethical approval. However, we will ensure that all included studies obtained the necessary ethical approvals.

**Table:** Status and timeline

Stage	Status	Timeline
Developing of the search strategy	Completed	
Search string validation	Completed	
Literature search	Completed	
Screening of records	Not started	25–30^th^ February 2025
Quality appraisal of included	Not started	1–7^th^ March 2025
Extraction of data	Not Started	8^th^ – 26^th^ March 2025
Synthesis of extracted data	Not started	27^th^ – 30^th^ March 2025
Writing of manuscript and Quality assessment using AMSTAR	Not started	1^st^ – 5^th^ April 2025

## Discussion

This protocol outlines a comprehensive approach to synthesizing evidence on SCA prevalence across Africa. Our systematic review and meta-analysis will address several key gaps in current knowledge while acknowledging certain limitations. By including studies published in any language, we aim to mitigate language bias and capture all relevant studies, thus providing a more accurate and representative picture of SCA prevalence. However, the varying quality of diagnostic methods may introduce heterogeneity that could affect our pooled estimates.

Despite these limitations, our study offers several strengths. The comprehensive search strategy, rigorous selection process, and robust statistical approach will provide the most complete synthesis of SCA prevalence data to date. The planned subgroup analyses will offer valuable insights into geographic and temporal trends, while our quality assessment approach ensures transparent evaluation of the evidence base.

The findings from this review will have significant implications for public health policy and clinical practice. Accurate prevalence estimates can inform resource allocation, guide screening programs, and help identify high-priority regions for intervention. Furthermore, understanding temporal trends may provide insights into the effectiveness of current control strategies and help predict future healthcare needs.

### Dissemination plans

The primary results will be published in a peer-reviewed journal following the PRISMA reporting guidelines.

## Supporting information

S1 File Preferred reporting items for systematic review and meta-analysis protocols (PRISMA-P) 2015 checklist. (PDF)

S2 File Search strings and results from PubMed, Google Scholar, Scopus and Bielefeld Academic Search Engine (BASE). (PDF)

S3 File Screening instructions for records searched. (PDF)

S4 File Form for records included and excluded with reasons for exclusion. (XLSX)

S5 File Data extraction form. (XLSX)
