# Supplementary material for: Prevalence of sickle cell anemia in Africa: A protocol for a meta-analysis of existing studies
Source: PLoS One. 2025 Apr 21;20(4):e0321535. doi: 10.1371/journal.pone.0321535 (PMC12011286; doi:10.1371/journal.pone.0321535)
Supplement: S1 File — (PDF) [file pone.0321535.s001.pdf]

Prevalence of sickle cell anemia in Africa: a meta-analysis protocol

PRISMA-P 2015 Checklist

This checklist has been adapted for use with protocol submissions to *Systematic Reviews* from Table 3 in Moher D et al: Preferred reporting items for systematic review and meta-analysis protocols (PRISMA-P) 2015 statement. *Systematic Reviews* 2015 **4**:1

Section/topic	#	Checklist item	Information reported		Line number(s)
			Yes	No	
**ADMINISTRATIVE INFORMATION**					
**Title**					
Identification	1a	Identify the report as a protocol of a systematic review	☒	☐	Line 1 - 2, page 1
Update	1b	If the protocol is for an update of a previous systematic review, identify as such	☐	☐	N/A
**Registration**	2	If registered, provide the name of the registry (e.g., PROSPERO) and registration number in the Abstract	☒	☐	Line 50 – 52, page 3
**Authors**					
Contact	3a	Provide name, institutional affiliation, and e-mail address of all protocol authors; provide physical mailing address of corresponding author	☒	☐	Line 4 – 15, page 1
Contributions	3b	Describe contributions of protocol authors and identify the guarantor of the review	☒	☐	Line 230 – 242, page 11
**Amendments**	4	If the protocol represents an amendment of a previously completed or published protocol, identify as such and list changes; otherwise, state plan for documenting important protocol amendments	☒	☐	Line 253 – 255, page 12
**Support**					
Sources	5a	Indicate sources of financial or other support for the review	☒	☐	Line 18, page 1
Sponsor	5b	Provide name for the review funder and/or sponsor	☒	☐	Line 20, page 1
Role of sponsor/funder	5c	Describe roles of funder(s), sponsor(s), and/or institution(s), if any, in developing the protocol	☒	☐	Line 20, page 1
**INTRODUCTION**					

BioMed Central
The Open Access Publisher

Section/topic	#	Checklist item	Information reported		Line number(s)
			Yes	No	
**Rationale**	6	Describe the rationale for the review in the context of what is already known	X		Line 24 – 29, page 2, line 53 – 85, page 3
**Objectives**	7	Provide an explicit statement of the question(s) the review will address with reference to participants, interventions, comparators, and outcomes (PICO)	X		Line 88 – 91, page 5
**METHODS**					
**Eligibility criteria**	8	Specify the study characteristics (e.g., PICO, study design, setting, time frame) and report characteristics (e.g., years considered, language, publication status) to be used as criteria for eligibility for the review	X		Line 96 – 118, page 5-6
**Information sources**	9	Describe all intended information sources (e.g., electronic databases, contact with study authors, trial registers, or other grey literature sources) with planned dates of coverage	X		Line 119 – 135, page 6 -7
**Search strategy**	10	Present draft of search strategy to be used for at least one electronic database, including planned limits, such that it could be repeated	X		line 313-page 14
STUDY RECORDS					
Data management	11a	Describe the mechanism(s) that will be used to manage records and data throughout the review	X		Lines 146 – 149, page 7
Selection process	11b	State the process that will be used for selecting studies (e.g., two independent reviewers) through each phase of the review (i.e., screening, eligibility, and inclusion in meta-analysis)	X		Lines 136 – 146, page 7
Data collection process	11c	Describe planned method of extracting data from reports (e.g., piloting forms, done independently, in duplicate), any processes for obtaining and confirming data from investigators	X		Lines 150 – 165, page 7-8
**Data items**	12	List and define all variables for which data will be sought (e.g., PICO items, funding sources), any pre-planned data assumptions and simplifications	X		Lines 150 – 165, page 7-8
**Outcomes and prioritization**	13	List and define all outcomes for which data will be sought, including prioritization of main and additional outcomes, with rationale	X		Lines 167– 181, page 8 -9
**Risk of bias in individual studies**	14	Describe anticipated methods for assessing risk of bias of individual studies, including whether this will be done at the outcome or study level, or both; state how this information will be used in data synthesis	X		Line 160– 165, page 8
DATA					
**Synthesis**	15a	Describe criteria under which study data will be quantitatively synthesized	X		Line 182– 195, page 9

Section/topic	#	Checklist item	Information reported		Line number(s)
			Yes	No	
	15b	If data are appropriate for quantitative synthesis, describe planned summary measures, methods of handling data, and methods of combining data from studies, including any planned exploration of consistency (e.g., I^2, Kendall's tau)	☒	☐	Line 182–195, page 9
	15c	Describe any proposed additional analyses (e.g., sensitivity or subgroup analyses, meta-regression)	☒	☐	Line 196 – 208, page 9 - 10
	15d	If quantitative synthesis is not appropriate, describe the type of summary planned	☒	☐	Line 182–195, page 9
**Meta-bias(es)**	16	Specify any planned assessment of meta-bias(es) (e.g., publication bias across studies, selective reporting within studies)	☒	☐	Line 190 – 195, page 9
**Confidence in cumulative evidence**	17	Describe how the strength of the body of evidence will be assessed (e.g., GRADE)	☒	☐	Line 209–204, page 9 - 10
