# Supplementary material for: Prevalence of sickle cell anemia in Africa: A protocol for a meta-analysis of existing studies
Source: PLoS One. 2025 Apr 21;20(4):e0321535. doi: 10.1371/journal.pone.0321535 (PMC12011286; doi:10.1371/journal.pone.0321535)
Supplement: S2 File — (PDF) [file pone.0321535.s002.pdf]

Prevalence of Sickle Cell Disease in Africa: Protocol for A Meta-Analysis

Search String

Table 1. Search string for PubMed database, from 1994 - 2024

Step	Search String	Pubmed Number of records
1.	"Sickle Cell Disease"[All Fields] OR "Sickle Cell Anaemia"[All Fields] OR "Sickle Cell "[All Fields]	25,837
2.	"Sickle Cell Disease"[All Fields] OR "Sickle Cell Anaemia"[All Fields] OR "Sickle Cell "[All Fields] AND ("prevalence"[All Fields] OR "epidemiology"[All Fields] OR "burden"[All Fields])	5,892
3.	"Sickle Cell Disease"[All Fields] OR "Sickle Cell Anaemia"[All Fields] OR "Sickle Cell"[All Fields] AND ("prevalence"[All Fields] OR "epidemiology"[All Fields] OR "burden"[All Fields]) AND ("Africa"[All Fields] OR "Uganda"[All Fields] OR "Nigeria"[All Fields] OR "Kenya"[All Fields] OR "Ghana"[All Fields] OR "South Africa"[All Fields] OR "Tanzania"[All Fields] OR "Ethiopia"[All Fields] OR "Democratic Republic of Congo"[All Fields] OR "Cameroon"[All Fields] OR "Ivory Coast"[All Fields] OR "Senegal"[All Fields] OR "Sudan"[All Fields] OR "Zambia"[All Fields] OR "Zimbabwe"[All Fields] OR "Malawi"[All Fields] OR "Mozambique"[All Fields] OR "Burkina Faso"[All Fields] OR "Mali"[All Fields] OR "Sierra Leone"[All Fields] OR "Benin"[All Fields] OR "Botswana"[All Fields] OR "Burundi"[All Fields] OR "Cape Verde"[All Fields] OR "Central African Republic"[All Fields] OR "Chad"[All Fields] OR "Comoros"[All Fields] OR "Djibouti"[All Fields] OR "Equatorial Guinea"[All Fields] OR "Eritrea"[All Fields] OR "Eswatini"[All Fields] OR "Gabon"[All Fields] OR "Gambia"[All Fields] OR "Guinea"[All Fields] OR "Guinea-Bissau"[All Fields] OR "Lesotho"[All Fields] OR "Liberia"[All Fields] OR "Madagascar"[All Fields] OR "Mauritania"[All Fields] OR "Mauritius"[All Fields] OR "Namibia"[All Fields] OR "Niger"[All Fields] OR "Rwanda"[All Fields] OR "Sao Tome and Principe"[All Fields] OR "Seychelles"[All Fields] OR "Somalia"[All Fields] OR "South Sudan"[All Fields] OR "Togo"[All Fields] OR "Western Sahara"[All Fields] OR "Algeria"[All Fields] OR "Angola"[All Fields] OR "Egypt"[All Fields] OR "Libya"[All Fields] OR "Morocco"[All Fields] OR "Tunisia"[All Fields])	1,982

Google Scholar

We utilized ***Harzing's Publish or Perish software*** [1]to search Google Scholar and Scopus. To address the 256-character limit in Publish or Perish, we optimized our search query by dividing it into multiple geographically organized batches. The search string was applied to the title field, covering the period from 1994 to 2024.

**Step**	**Search string**	**Google Scholar Number of records**	**Scopus Number of records**
1.	"Sickle Cell Disease" OR "Sickle Cell Anaemia" OR "Sickle Cell" AND ("prevalence" OR "epidemiology" OR "burden") AND ("Uganda" OR "Nigeria" OR "Kenya" OR "Ghana" OR "South Africa")	166	52
2.	"Sickle Cell Disease" OR "Sickle Cell Anaemia" OR "Sickle Cell" AND ("prevalence" OR "epidemiology" OR "burden") AND ("Tanzania" OR "Ethiopia" OR "DR Congo" OR "Cameroon" OR "Ivory Coast")	34	19
3.	"Sickle Cell Disease" OR "Sickle Cell Anaemia" OR "Sickle Cell" AND ("prevalence" OR "epidemiology" OR "burden") AND ("Senegal" OR "Sudan" OR "Zambia" OR "Zimbabwe" OR "Malawi")	22	4
4.	"Sickle Cell Disease" OR "Sickle Cell Anaemia" OR "Sickle Cell" AND ("prevalence" OR "epidemiology" OR "burden") AND ("Mozambique" OR "Burkina Faso" OR "Mali" OR "Sierra Leone")	5	4
5.	"Sickle Cell Disease" OR "Sickle Cell Anaemia" OR "Sickle Cell" AND ("prevalence" OR	15	4

	"epidemiology" OR "burden") AND ("Benin" OR "Botswana" OR "Burundi" OR "Cape Verde" OR "Central African Republic")		
6.	"Sickle Cell Disease" OR "Sickle Cell Anaemia" OR "Sickle Cell" AND ("prevalence" OR "epidemiology" OR "burden") AND ("Chad" OR "Comoros" OR "Djibouti" OR "Equatorial Guinea" OR "Eritrea")	0	0
7.	"Sickle Cell Disease" OR "Sickle Cell Anaemia" OR "Sickle Cell" AND ("prevalence" OR "epidemiology" OR "burden") AND ("Eswatini" OR "Gabon" OR "Gambia" OR "Guinea" OR "Guinea-Bissau")	4	2
8.	"Sickle Cell Disease" OR "Sickle Cell Anaemia" OR "Sickle Cell" AND ("prevalence" OR "epidemiology" OR "burden") AND ("Lesotho" OR "Liberia" OR "Madagascar" OR "Mauritania" OR "Mauritius")	3	8
9.	"Sickle Cell Disease" OR "Sickle Cell Anaemia" OR "Sickle Cell" AND ("prevalence" OR "epidemiology" OR "burden") AND ("Namibia" OR "Niger" OR "Rwanda" OR "Sao Tome and Principe" OR "Seychelles")	3	1
10.	"Sickle Cell Disease" OR "Sickle Cell Anaemia" OR "Sickle Cell" AND ("prevalence" OR "epidemiology" OR "burden") AND ("Somalia" OR "South Sudan" OR "Togo" OR "Western Sahara" OR "Algeria")	2	2
11.	"Sickle Cell Disease" OR "Sickle Cell Anaemia" OR "Sickle Cell" AND ("prevalence" OR "epidemiology" OR "burden") AND ("Angola" OR "Egypt" OR "Libya" OR "Morocco" OR "Tunisia")	3	2

Bielefeld Academic Search Engine (BASE)

The search was conducted using the basic search interface of the Bielefeld Academic Search Engine (BASE), focusing on titles related to sickle cell disease, its prevalence, epidemiology, and burden, with a specific emphasis on 54 African countries. The query yielded a total of 1305 records, which were categorized into various types of academic contributions. These included 1123 articles, 83 texts, 53 reviews, 32 reports, and 32 theses. In addition, the search results encompassed 18 unknown records, 17 other non-articles, 16 master's theses, 12 doctoral and postdoctoral theses, 10 conference objects, 7 datasets, 4 book parts, 2 bachelor's theses, 1 book, 1 manuscript, and 1 still image.

Step	Search string	Number of records
1.	("sickle cell disease" OR "sickle cell anaemia" OR "sickle cell disorder") AND ("prevalence" OR "epidemiology" OR "burden") AND ("Africa" OR "Uganda" OR "Nigeria" OR "Kenya" OR "Ghana" OR "South Africa" OR "Tanzania" OR "Ethiopia" OR "Democratic Republic of Congo" OR "Cameroon" OR "Ivory Coast" OR "Senegal" OR "Sudan" OR "Zambia" OR "Zimbabwe" OR "Malawi" OR "Mozambique" OR "Burkina Faso" OR "Mali" OR "Sierra Leone" OR "Benin" OR "Botswana" OR "Burundi" OR "Cape Verde" OR "Central African Republic" OR "Chad" OR "Comoros" OR "Djibouti" OR "Equatorial Guinea" OR "Eritrea" OR "Eswatini" OR "Gabon" OR "Gambia" OR "Guinea" OR "Guinea-Bissau" OR "Lesotho" OR "Liberia" OR "Madagascar" OR "Mauritania" OR "Mauritius" OR "Namibia" OR "Niger" OR "Rwanda" OR "Sao Tome and Principe" OR "Seychelles" OR "Somalia" OR "South Sudan" OR "Togo" OR "Western Sahara" OR "Algeria" OR "Angola" OR "Libya" OR "Morocco" OR "Tunisia")	1,305
2.	Search limit 1994 - 2024	1, 277
