# Supplementary material for: Prevalence of sickle cell anemia in Africa: A protocol for a meta-analysis of existing studies
Source: PLoS One. 2025 Apr 21;20(4):e0321535. doi: 10.1371/journal.pone.0321535 (PMC12011286; doi:10.1371/journal.pone.0321535)
Supplement: S3 File — (PDF) [file pone.0321535.s003.pdf]

<u>Prevalence of Sickle cell in African, a protocol for a meta-analysis</u>

<u>Screener Instructions</u>

1. Import all the RIS files for the searched for records to Zotero citations manager
2. Use the duplicate items function to remove duplicate records by merging
3. Familiarize with the eligibility criteria and use them as basis for screening decisions
4. Using Zotero, effectively use the search filters "Africa" and names of all 54 African countries to identify studies conducted within Africa. Search these terms across all fields and tags ("Algeria", "Angola", "Benin", "Botswana", "Burkina Faso", "Burundi", "Cabo Verde", "Cameroon", "Central African Republic", "Chad", "Comoros", "Congo (Republic of the Congo)", "Congo (Democratic Republic of the Congo)", "Djibouti", "Egypt", "Equatorial Guinea", "Eritrea", "Eswatini", "Ethiopia", "Gabon", "Gambia", "Ghana", "Guinea", "Guinea-Bissau", "Ivory Coast", "Kenya", "Lesotho", "Liberia", "Libya", "Madagascar", "Malawi", "Mali", "Mauritania", "Mauritius", "Morocco", "Mozambique", "Namibia", "Niger", "Nigeria", "Rwanda", "São Tomé and Príncipe", "Senegal", "Seychelles", "Sierra Leone", "Somalia", "South Africa", "South Sudan", "Sudan", "Togo", "Tunisia", "Uganda", "Zambia", "Zimbabwe")
5. Read titles and abstracts carefully. Assess whether the records meet the eligibility criteria (Apply other developed screening questions consistently for all records to assess eligibility, supplementary information, **S2 File**)
6. Assess the full-text articles that pass the initial screening against all eligibility criteria. Ensure both sample size, frequency of sickle cell disease cases, and method used to test to SCD are provided in the text.
7. For every record, note whether it was included or excluded. For excluded records, provide reasons for exclusion based on the criteria (Supplementary information, **S3 File**)
8. Highlight records where disagreements occur during the title, abstract, or full-text screening.
9. Use Cohen's kappa statistic to measure the inter-rater agreement during the title and abstract screening phase
10. Participate in consensus discussions to finalize inclusion or exclusion decisions
